# Implications of Intrathecal Chemotherapy for Anaesthesiologists: A Brief Review

**DOI:** 10.1155/2016/3759845

**Published:** 2016-03-31

**Authors:** Abhijit Nair

**Affiliations:** Department of Anaesthesiology, Basavatarakam Indo-American Cancer Institute and Research Centre, Hyderabad 500034, India

## Abstract

Intrathecal chemotherapy is routinely prescribed in medical oncology practice, either for prophylaxis or for treatment of leptomeningeal disease due to a primary haematological disease or a metastatic disease due to any other malignancy. As these groups of patients are coagulopathic either because of the disease per se or due to systemic chemotherapy, lumbar puncture in them is considered challenging and is expected to be performed by an anaesthesiologist because of their expertise in this procedure. However, the challenge is not only in performing the lumbar puncture safely but also in dealing with other issues like explaining and handling complications that can happen either due to the drug injected intrathecally or due to a neurodeficit occurring either due to the underlying coagulopathy or due to the progression of leptomeningeal disease.

## 1. Introduction

Anaesthesiologists are trained in performing lumbar puncture (LP) during their postgraduation training programme with a spinal needle to perform spinal anaesthesia for a variety of surgeries involving lower abdomen and lower extremities. In their routine practice in medical colleges or corporate hospitals or as freelance anaesthesiologist working at nursing homes, they perform spinal anaesthesia very frequently. Therefore they are considered as experts in lumbar puncture and are requested to perform lumbar puncture for various specialities like neurology (CSF analysis, CSF pressure monitoring), radiology (myelography), and medical oncology (intrathecal chemotherapeutic agents) especially when it is difficult for the other speciality physicians to perform.

Anaesthesiologists usually oblige the specialists by performing a lumbar puncture and injecting the drug intrathecally prescribed by them after reviewing the coagulation profile (platelet count, prothrombin time, and activated partial thromboplastin time) and after taking an informed consent. Literature describes life-threatening events happening after intrathecal chemotherapeutic injections [1]. What is difficult to answer is that how much liable the anaesthesiologist is because they punctured the dura for injecting the drug as requested by a specialist for a diagnostic/therapeutic purpose. In the court of law, both specialists could be equally responsible as one has prescribed (the one who wants lumbar puncture) and the other one who performed the procedure (the anaesthesiologist). The purpose of this article is to briefly know about the drugs that are injected intrathecally in medical oncology, to know the possible complications after intrathecal (IT) chemotherapeutic injections and the possible ways where the anaesthesiologist can avoid a medicolegal issue. We have restricted the discussion to the most commonly used drugs which are injected intrathecally in the practice of medical oncology as a part of chemotherapy regime. Methotrexate, cytarabine, thiotepa, trastuzumab, and corticosteroids are the drugs administered intrathecally for prophylaxis or palliative treatment of meningeal involvement due to haematological malignancies, primary brain malignancies, or metastatic disease (usually breast malignancies). In practice, methotrexate and cytarabine are the drugs frequently prescribed for intrathecal administration.

*(1) Methotrexate.* Methotrexate is a tetrahydrofolate dehydrogenase inhibitor which prevents tetrahydrofolate formation required for thymidylate synthesis, which is an essential component of DNA (deoxyribonucleic acid). It has antineoplastic, antimetabolite, and immunosuppressive properties [2]. IT methotrexate is usually prescribed prophylactically in patients with leukemia or lymphoma where there is a high risk of central nervous system (CNS) involvement. Therapeutically, IT injection is done in patients with proven or suspected leptomeningeal carcinomatosis. 6 to 15 mg methotrexate is usually injected intrathecally. IT methotrexate is now preferred over cranial irradiation for prophylaxis and treatment of central nervous system leukemia as radiation was associated with secondary malignancies, growth retardation, and developmental delay in pediatric patients [3].

Complications of intrathecal methotrexate are headache, seizures, coma, neurodeficit, aphasia, and cardiovascular compromise. The neurotoxicity associated with IT methotrexate are described as acute, subacute, and chronic [4]. The proposed mechanism of neurotoxicity due to IT methotrexate is mediated by adenosine. Methotrexate inhibits dihydrofolate reductase which leads to increased concentration of adenosine and homocysteine. Adenosine leads to cerebral vasodilatation, slows neurotransmitter release at presynaptic junction, and slows neuronal discharge due to modified postsynaptic response. Neurotoxicity is more often seen in patients where it is administered for treatment as compared to prophylaxis as in established CNS involvement owing to bulky disease; the drug does not spread well leading to toxic concentration of drug at the surrounding tissue [5]. Once detected, the management includes supportive care and measures to drain CSF like lumbar puncture, systemic corticosteroids, and leucovorin. MRI with diffusion usually shows restricted contrast diffusion within white matter. Aminophylline being a competitive antagonist of adenosine is used to treat neurotoxicity mediated by methotrexate at a dose of 2–5 mg/kg. Carboxypeptidase G2 is an enzyme that metabolizes methotrexate when given intravenously to its inactive metabolites 4-deoxy-4-deamino-N-methyl-pteroic acid and glutamate [6].

*(2) Cytarabine.* Cytarabine is a pyrimidine nucleoside analog which inhibits the synthesis of DNA at the S phase of the cell cycle. Like methotrexate, it has antimetabolite, antineoplastic, and immunosuppressive properties [7, 8]. Intrathecal liposomal cytarabine is indicated for the prophylaxis and treatment of meningeal leukemia, brain tumors (medulloblastoma, meningioma, neuro-ectodermal tumors, germ cell tumors, and oligodendroglioma), lymphoma, and disseminated malignancies (breast) [9]. The liposomal variant of cytarabine is prepared in the form of biodegradable, lipid base particles. This alteration prolongs the exposure of cytarabine in the cerebrospinal fluid (CSF). The liposomal formulation is known to exert its effect in CSF for a week in children and almost 2 weeks in adults whereas the conventional cytarabine exerts its effect in CSF for less than 24 hours. The recommended dose of liposomal cytarabine is up to 50 mg in adults and 35 mg in children [10]. Chemical arachnoiditis is a frequently encountered problem with intrathecal liposomal arachnoiditis. It is characterised by headache, back pain, fever, nausea, and vomiting. This problem usually is minimised by administration of systemic dexamethasone [11–13]. Life-threatening neurotoxicity was noticed by Jabbour et al. when systemic and intrathecal cytarabine had an overlap in high risk patients. Neurotoxicity manifested as cauda equina syndrome, encephalopathy, seizures, and pseudotumor cerebri [14]. Yoon et al. reported a case involving diffuse cerebral vasospasm and acute cerebral infarct in a 7-year-old child diagnosed as ALL, after intrathecal cytarabine [15]. Butto et al. reported death in a 56-year-old female patient who was treated with intrathecal cytarabine for leptomeningeal spread due to breast cancer. The patient developed progressive loss of consciousness leading to loss of brain stem function and presented with diffuse cerebral oedema on CT scan due to fulminant chemical ventriculomeningitis [16].

*(3) Trastuzumab.* Trastuzumab is a monoclonal IgG1 antibody usually used to treat metastatic breast carcinoma especially the tumors which overexpress HER2/neu protein. In patients who develop leptomeningeal metastasis (involvement of pia and arachnoid matter), the usual intravenous dose does not help because of poor cerebrospinal penetration. In such cases, intrathecal trastuzumab is advocated along with systemic trastuzumab [17]. The dosing and the duration is controversial. Intrathecal methotrexate and/or cytarabine are also administered intrathecally along with trastuzumab as described in some case series [18]. Trastuzumab was used intrathecally in combination with another monoclonal antibody rituximab in patients diagnosed with leptomeningeal carcinomatosis [19].

*(4) Thiotepa.* Thiotepa or N,N′,N′′-triethylenethiophosphoramide is an alkylating agent with broad spectrum antineoplastic activity. Intrathecal thiotepa has been used in combination with trastuzumab in patients with breast cancer with leptomeningeal spread [20, 21]. Although it is approved for intrathecal use in meningeal carcinomatosis, thiotepa is not as popular as compared to methotrexate and cytarabine possibly because large volume studies are not available describing its use. Comte et al. publish a retrospective study describing survival and prognostic factors in breast cancer patients presenting with meningeal carcinomatosis who were treated with intrathecal thiotepa. The authors claimed it to be the largest retrospective cohort of breast cancer patients in whom IT thiotepa was used. The study involved 66 patients who received thiotepa as either the first line or the second line of drug. The results of the study revealed that although patients benefitted from its use even when used as a second line drug, the median survival was short [22].

## 2. Postprocedural Care and Observation of High Risk Injection

Patients in whom dural puncture took several attempts and patients who came for the procedure after a platelet transfusion are the high risk ones. Usually these patients are hospitalized; therefore, they can be monitored. The nurse involved in the care of such patients should be instructed to inform the primary physician or the anaesthesiologist if patient develops a new onset lower limb weakness, severe headache, altered mentation, or seizures. A multidisciplinary approach should be executed in such physicians. After examining the patient thoroughly, a neurophysician and a neurosurgeon should be consulted. If necessary, relevant imaging like a CT scan or an MRI should be considered to rule out hematoma in the neuraxis. Such patients should be transferred to an intensive care unit for monitoring.

*How to Avoid a Lawsuit [23]?* A simple lumbar puncture in a cancer patient leading to complications described related to the medication injected or because of a new onset neurodeficit can have serious medicolegal implications if certain simple, basic steps are not followed or casually ignored. We have described some points that should be addressed seriously when involved in such injections.

(1) Informed consent: the consent should be preferably written with all unexpected problems explained to patient and/or family members. Introduce yourself to the patient and family members and tell them why you are involved in the medical management. Sometimes, when the patient or family members get apprehensive by seeing a new doctor around, it is better to be introduced by the treating physician.

(2) It is better to perform a comprehensive neurological examination and if any neurodeficits are encountered, it should be documented in bold letters. Patient/family members should be told about it if they are not knowing it prior to the procedure.

(3) Document everything (platelet count, attempts taken for the procedure, and size of LP needle). If it is not written, it is not done; that is the dictum.

(4) If multiple attempts are made or SDP/RDP cover is not given for a low platelet count, follow up the patient in the ward and document your visit. Most of the time, the IT injection is offered on an outpatient basis. In such situation, making a telephone call and enquiring about any neurological issues not only keeps the anaesthesiologist informed about the patient's condition but also helps to build a rapport.

(5) In case of any adverse event or a complication, appropriate consultation (neurologist, neurosurgery) should be asked for and, if necessary, relevant imaging should be advised (CT scan, MRI).

## 3. Published Guidelines and their Application in Medical Oncology **[24–26]**

Most of the time the medical oncology patients who require prophylactic or therapeutic lumbar puncture have a low platelet count due to previous or ongoing systemic chemotherapeutic agents. When platelet count is less than 20,000/cu·mm, the patients are transfused with single donor platelets (SDP) or random donor platelets (RDP). But when platelet count is around or more than 50,000 but less than 75,000/cu·mm, the patients are not transfused even when they have a lumbar puncture planned for IT chemotherapy. But ASRA (American Society of Regional Anesthesia) guidelines have set a cutoff of a platelet count of 75,000/cu·mm for a safe central neuraxial block so as to avoid a possible spinal/epidural hematoma leading to neurodeficits. This is where the conflict starts. The anaesthesiologist is called for help in case of a previously difficult LP or an LP which this time is difficult to the nonanaesthesiology colleague who has a platelet count much lower than the cutoff recommended by ASRA and there is no SDP/RDP transfusion given to this patient. The oncologist/physician would have given the IT medication if the LP was successful. Now that it is getting difficult, how should we proceed?

Let us consider another situation. The anaesthesiologist, in order to help a physician in getting a lumbar puncture, obliges and the patient develops a neurodeficit (e.g., hemiparesis). Whom should the patient blame? Unfortunately, we do not have time to explain the possible complications due to thrombocytopenia or due to the procedure per se when we reach the scene to help a person who was unsuccessful in performing an LP about. We assume or it is quite likely that the oncologist must have explained drug related problems to the patient/family members (e.g., neurotoxicity). The patient/family members will not forget the new person who managed an LP who is otherwise not seen during his or her treatment. In case of a child, the parents will notice that the puncture was done by a different person who is not directly involved in the treatment of the patient. However, in situations where the help of an anaesthesiologist is taken in case of a previously difficult LP, there is time to take a proper informed consent and explain possible complications related to thrombocytopenia to the patient. The anaesthesiologist can actually request the oncologist for a platelet transfusion (single donor or random donor) and get the LP done under the cover of the transfusion, which is usually done if the count is less than 20,000/cu·mm.

In case of metastatic leptomeningeal disease, the prognosis is usually not good. There is always a possibility of sudden, new onset neurodeficits due to progressive nature of metastatic disease. Patients who have confirmed leptomeningeal metastases are usually well counselled by the treating oncologist. However, if a lumbar puncture is done in situation with preexisting low platelet count not adequately covered by an SDP/RDP transfusion, it is difficult to prove who is to blame (the disease or the procedure, i.e., LP) in case the patient develops a new onset neurodeficit after the injection.

As a specialist, it is difficult to refuse such procedures because an anaesthesiologist is looked upon as an expert in doing LP. However, evidence based approach and a detailed discussion with the oncologist and the patient's family will make it a hassle-free procedure.

## 4. Use of Ommaya Reservoir for Intraventricular Injection

Ommaya reservoir is an intraventricular catheter system that is surgically implanted for either aspiration of cerebrospinal fluid or injecting drugs intrathecally. The reservoir can be placed either by an open surgical technique or by using navigation system [27–29]. The paper published by Peyrl et al. after a 20-year experience with 5472 intraventricular drug injections in 98 patients with brain tumors confirmed the feasibility and safety of Ommaya reservoir in administering intraventricular injections directly [30]. However, as expected, they suggested to train the personnel involved in handling the device with asepsis. The reservoir placement requires general anaesthesia for the patients. By following standard principles of neuroanesthesia, that is, attenuating hemodynamic response to intubation and extubation response, avoiding increase in intracranial pressure, and avoiding hypertension and hypercarbia; the reservoir placement can be done uneventfully. The study conducted by Zhang showed good outcomes in 45 patients who received chemotherapy for intracranial tumors via Ommaya reservoir [31].

## 5. Conclusion

The anaesthesiologist involved in performing intrathecal chemotherapeutic indications should know the disease of the patient, the drug which is prescribed for chemotherapy, and the possible adverse events associated with the use of those drugs. A preanaesthesia evaluation should be insisted before such procedures and the details of disease, previous chemotherapy, haematological investigations, and neurodeficits (if any) should be documented. Informed consent should be taken from patient or family member in case of a minor patient and possible complications should be explained in detail. These injections are electively done; therefore even if an anaesthesiologist spends 15 minutes in speaking to the patient or family member, a rapport can be developed with them. Documentation of the procedure details should be always done.
